# Hemosuccus Pancreaticus: A Rare Bleeding Pseudoaneurysm of the Inferior Pancreaticoduodenal Artery Treated with Embolization

**DOI:** 10.1155/2018/2354169

**Published:** 2018-09-02

**Authors:** Luke L. Wang, Zachary M. Bauman

**Affiliations:** University of Nebraska Medical Center, Trauma, Emergency General Surgery, Critical Care Surgery, Omaha, USA

## Abstract

Hemosuccus pancreaticus is a very rare cause of gastrointestinal bleeding and can be life-threatening if not managed appropriately. Still thought to be a surgical problem, advances in medical therapy now afford these patients the opportunity to undergo less-invasive angiography techniques to manage this illness when it occurs. Here, we present a case of hemosuccus pancreaticus safely managed with liquid N-butyl-2-cyanoacrylate embolization.

## 1. Introduction

Hemosuccus pancreaticus is an especially rare cause of gastrointestinal bleeding. It is defined as bleeding into the duodenum from the Vater papilla through the route of the pancreatic duct, also known as Wirsungorrhaghia [1–3]. Most cases (60–80%) are due strictly to a pancreatic origin which mostly include pancreatic malformations and ductal wall ulcers [1–3]. Rarely (incidence of 6–17%), hemosuccus pancreaticus is the result of a ruptured peripancreatic pseudoaneurysm directly into a communicating pancreatic duct, likely through a pancreatic pseudocyst [1, 4, 5].

Pancreatic arterial pseudoaneurysms are infrequent complications of both acute and chronic pancreatitis [4]. These pseudoaneurysms are thought to arise from pancreatic enzyme-mediated destruction of the vessel wall [4]. Although unlikely, acute hemorrhage from these ruptured pseudoaneurysms can be fatal [4]. In a review of 98 cases of hemorrhage from pancreatic pseudocysts and pseudoaneurysms from an identifiable bleeding vessel in the setting of pancreatitis, 45% arose from the splenic artery, 17% from the gastroduodenal artery, 11% from the inferior pancreaticoduodenal artery (IPDA), 5% from the superior pancreaticoduodenal artery, and 22% from other vessels [6]. The diagnosis of hemosuccus pancreaticus is based on clinical exam and past medical history of pancreatitis. Furthermore, contrast-enhanced computed tomography (CT) can be used to identify a pancreatic and/or peripancreatic pseudoaneurysm followed by confirmation utilizing angiography or intraoperatively in emergencies [1–4]. Recently, various groups have employed embolization in lieu of arterial ligation and/or pancreatectomy in managing bleeding pancreatic pseudoaneurysms [4, 5, 7]. We add to the literature a rare case of hemosuccus pancreaticus from an acute hemorrhagic IPDA pseudoaneurysm treated with embolization.

## 2. Case Report

**Figure 1:**
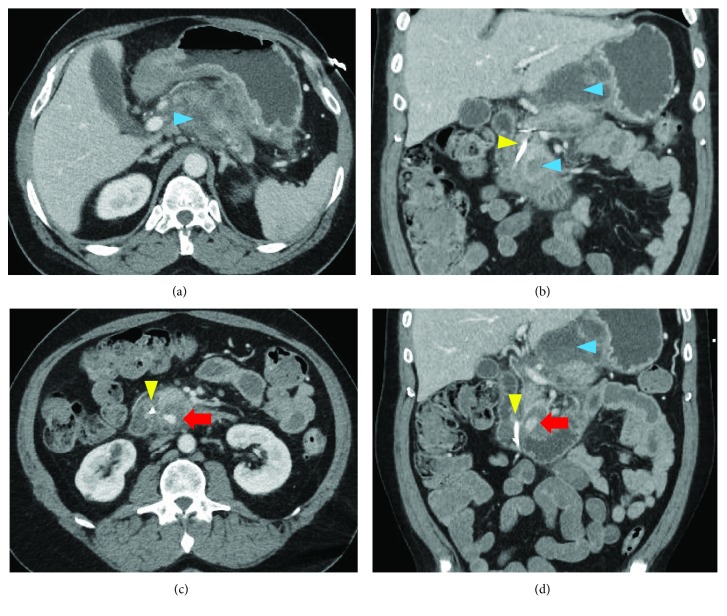
CT showing pancreas with necrotic fluid collections on axial (a) and coronal (b) sections. Contrast extravasation into the IPDA pseudoaneurysm (red arrows) on axial (c) and coronal (d) sections. Yellow arrowheads point to pancreatic duct stent. Blue arrowheads point to pancreatic fluid collections.

A 49-year-old male with a history of alcoholism, chronic pancreatitis with pancreatic duct stenting, and newly diagnosed diabetes mellitus type II was admitted to our hospital for abdominal pain, melena, nausea, and vomiting in the setting of necrotizing pancreatitis and blood loss anemia with a hemoglobin count of 6.6 g/dL. CT showed pancreatic tail atrophy from prior necrosis, a new area of necrosis measuring 7.1 × 4.9 × 4.9 cm at the superior aspect of the pancreas body and a new 2.3 × 2.2 × 2.8 cm fluid collection inferior-posterior to the uncinate process. (The latter fluid collection communicated with the second portion of the duodenum on CT and would later be identified as the IPDA pseudoaneurysm.) There was extravasation of contrast within this latter fluid collection, suggestive of active bleeding (Figure 1). During this time, the patient responded well to volume resuscitation and remained hemodynamically stable. It was felt that this bleeding was likely the result of inflammatory erosion into an artery. Given the patient's stability, the decision was made to attempt angiography with embolization. Surgical correction was considered at this time but ultimately would be more morbid and challenging given the degree of inflammation with his necrotizing pancreatitis.

**Figure 2:**
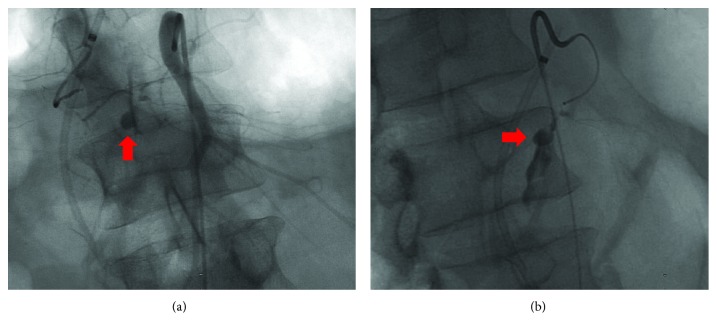
Angiography. (a) Superior mesenteric arteriogram showing pseudoaneurysm at the IPDA (red arrow). (b) Selective catheterization of the IPDA showing active extravasation into the pancreatic duct from the IPDA pseudoaneurysm (red arrow).

A superior mesenteric arteriogram showed a pseudoaneurysm arising from the IPDA with intermittent hemorrhage (Figure 2). The microcatheter and wire could not be advanced past the pseudoaneurysm or into the pseudoaneurysm without buckling and displacing the microcatheter from the IPDA. In addition, the feeding artery from the superior pancreaticoduodenal artery could not be seen from celiac catheterization. Placing a microcoil in the proximal IPDA would have blocked the access to the pseudoaneurysm for definitive treatment. Thus, the decision was made to embolize the pseudoaneurysm with liquid N-butyl-2-cyanoacrylate (NBCA). Follow-up digital subtraction arteriography confirmed successful occlusion of the pseudoaneurysm and IPDA.

Thereafter, the patient was initially admitted to the surgical intensive care unit for close monitoring. He did have persistent melanous bowel movements, requiring 2 units immediately, one unit at 34 hours, and 4 units at 57 hours post embolization. His melanotic stools resolved 5 days post embolization. Repeat CT angiogram on hospital day 5 showed complete occlusion of the IPDA. A small amount of gas was noted in a thick-walled duodenum along the region of prior embolization, which was concerning for ischemic necrosis without perforation. Upper endoscopy the following day showed normal stomach with mild duodenitis and no evidence of necrosis or bleeding. He was discharged in stable condition on hospital day 7, tolerating a regular diet without blood in his stool. He was doing well at his 1-month follow-up appointment and denying abdominal pain or melanotic bowel movements.

## 3. Discussion

It has been proposed that hemosuccus pancreaticus secondary to a hemorrhagic pancreatic pseudoaneurysm should be suspected in any patient with pancreatitis and persistent or increased abdominal pain in the setting of gastrointestinal bleeding, hemodynamic instability, or a drop in hematocrit [1–3, 5]. It has been reported on various occasions that hemorrhage may recur even after successful embolization [5, 6]. In one retrospective series of 10 patients with bleeding pancreatic pseudoaneurysms in the setting of acute or chronic pancreatitis, one patient died from massive hemorrhage that occurred 7 days after microcoil embolization [5]. The authors proposed that embolization should be a temporizing measure before definitive surgery [5]. In our case, NBCA was employed over microcoil for technical considerations outlined above. It is unclear if NBCA attenuates the risk of post embolization rebleeding. Rebleeding after NBCA has been documented at 11, 33, and 49 days post embolization in one retrospective series (2 cases of IPDA pseudoaneurysm) and was successfully managed with repeat embolization in all cases [7].

We believe operative therapy is necessary for hemosuccus pancreaticus secondary to bleeding IPDA pseudoaneurysms (or any bleeding peripancreatic pseudoaneurysms for that matter) not responsive to initial embolization or in the setting of hemodynamic instability. Due to the rarity of hemosuccus pancreaticus, there is no current consensus in the literature on the best surgical approach, which can vary according to pseudoaneurysm location. In general, intracystic suture ligation with proximal and/or distal arterial ligation has been employed to control hemorrhage when surgery is warranted [4, 6]. An alternative surgical approach is partial pancreatectomy, which was shown in one case series having a lower rate of rebleeding compared to ligation [4]. Splenectomy with distal pancreatectomy may be required for hemorrhagic splenic artery aneurysms [4, 6]. Total or partial gastrectomy or hemicolectomy has also been performed for bleeding into the stomach or colon. Pancreaticoduodenectomy and necrosectomy are a surgical extreme that may be required based on the severity of the disease process [4, 5].

Our patient's persistent melanotic stools draw attention to the ongoing risk of rebleeding in patients after pancreatic pseudoaneurysm embolization. Regardless of whether definitive surgery is required after successful initial embolization, a long-term follow-up for these patients is warranted.
